# No fire without smoke (particles)

**DOI:** 10.7554/eLife.74331

**Published:** 2021-11-17

**Authors:** Stephanie M Holm, John Balmes

**Affiliations:** 1 Department of Medicine, University of California, San Francisco San Francisco United States

**Keywords:** child health, wildfire, climate change, maternal health, Human

## Abstract

Pollution from landscape fires, which are increasing with climate change, leads to babies being born with lower birthweights in low- and middle-income countries.

**Related research article** Li J, Guan T, Guo Q, Geng G, Wang H, Guo F, Li J, Xue T. 2021. Exposure to landscape fire smoke reduced birthweight in low- and middle-income countries: findings from a siblings-matched case-control study. *eLife* **10**:e69298. doi: 10.7554/eLife.69298

Babies who are born with low birthweight (less than 2,500 g) or very low birthweight (less than 1,500g) are at risk of early postnatal complications, developmental problems during childhood, and additional diseases throughout their lifetime. Low birthweight is unfortunately very common, especially in low- and middle-income countries, where over 90% of low birth-weight babies are born (Blencowe et al., 2019). Because low birthweight is a global public health issue of major importance, the World Health Organization has made reducing its incidence one of its 2025 global targets. This is also a United Nations Sustainable Development Goal (World Health Organization, 2014).

While poverty – with associated malnutrition and infections – is likely the greatest cause of low birthweight in low- and middle-income countries, exposure to toxic agents in the environment can also contribute. Exposure to air pollution has previously been linked to both low birthweight and premature births (Li et al., 2017), especially air pollution due to particulate matter measuring less than 2.5 microns in diameter (PM_2.5_). By comparison, the diameter of a human hair is about 60 microns. PM_2.5_ particles are small enough to be inhaled deep into the lungs, where they can injure the delicate air sacs, and even cross into the bloodstream and cause issues in other organs. Most of the research on exposure to PM_2.5_ and low birthweight focuses on urban air pollution rather than on landscape fire smoke, which includes wildfires, deforestation fires, and burning of agricultural crop residues.

Climate change and deliberate deforestation have both contributed to a marked increase in large landscape fires in recent years (Hantson et al., 2017). The smoke from these fires contains many toxic agents, including PM_2.5_, and can lead to poor air quality over wide regions downwind from the fires. Now in eLife, Tao Xue from the Peking University Health Science Center and colleagues from the Peking Union Medical College, the University of Science and Technology Beijing, Tsinghua University and Zheijang University – with Jiajianghui Li and Tianjia Guan as joint first authors – report on how PM_2.5_ from landscape fires contributes to low birthweight in low and middle-income countries (Li et al., 2021).

Li et al., 2021 collected information on almost 228,000 babies born between 2000 and 2014 in 54 low- and middle-income countries. Birthweight data and individual characteristics came from demographic and health Surveys. Exposure to landscape fire PM_2.5_ during pregnancy was assessed through a sophisticated approach known as a chemical transport model (Xue et al., 2021), which was evaluated by comparing it to another well-known satellite-based approach (van Donkelaar et al., 2016). A chemical transport model uses information about pollutant emissions and atmospheric conditions to model the levels of different air pollutants, while the satellite approach uses satellite imagery to approximate the concentration of particles in the air. Li et al. then used a study design called a ‘sibling-matched case-control’, where babies are compared to their less exposed siblings, to study the association between landscape fire PM_2.5_ and birthweight, including the specific categories of low and very low birthweight. This design allows researchers to control for many of the complex factors that affect birthweight, including genetics, socioeconomic status, and quality of local health care, which can be difficult to measure.

Li et al. found that exposure to landscape PM_2.5_ during pregnancy meant babies had lower birthweights on average, and also that more babies met the criteria for low birthweight and very low birthweight (Figure 1). The association was stronger for female babies, first-born babies, and babies born to unemployed mothers. Moreover, babies born to families that had already had children with low average birthweights were most at risk. Li et al. controlled for multiple factors that might have led to bias in their results, including maternal age, child sex, multiple births, non-landscape fire PM_2.5_, birth order, temperature, humidity, month and year of birth, and country. Much of the effect of landscape fire PM_2.5_ was found in Sub-Saharan Africa, where approximately half of the babies were born, suggesting that other factors may have larger contributions to lower birthweights in other regions.

**Figure 1.**
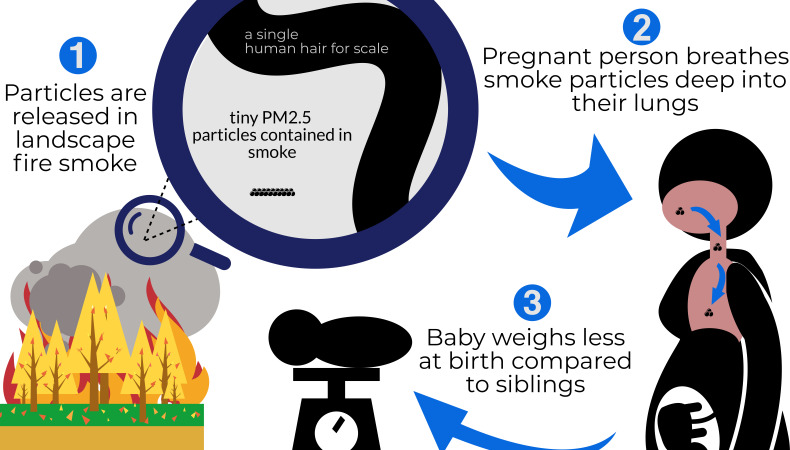
Schematic representation of Li et al.'s findings. From the left, clockwise: (1) Landscape fires release particulate matter into the air, including particles measuring less than 2.5 microns in diameter (PM_2.5_). A magnifying glass shows how much smaller these particles are compared to the width of a strand of human hair (these particles are 2.5 microns or smaller; a hair is 60 microns) (2) Breathing in these tiny particles during pregnancy draws the particles deep into the lungs, where they lead to irritation and inflammation and enter the bloodstream affecting other organs, as well as the growing fetus. (3) When the baby is born, it weighs less than its siblings, who were exposed to lower levels of PM_2.5_ during pregnancy.

This study has many strengths, including a large study population, information on individual maternal characteristics, a sophisticated approach to assessing landscape fire PM_2.5_ exposure, and careful data analysis. The results are consistent with several smaller studies from high-income countries (Holstius et al., 2012; Cândido da Silva et al., 2014; Abdo et al., 2019). Perhaps the most impactful contribution of Li et al. is the focus on exposure to landscape fire PM_2.5_ in low and middle-income countries.

Despite its strengths, the study has several limitations. The survey data used did not have information on the date of conception, so a uniform nine month pregnancy period was used in the analysis. However, babies with low birthweights sometimes have shorter gestations, which would mean the exposure to landscape fire PM_2.5_ could have been underestimated for these babies. Another limitation is that the study relies on the mothers’ memory of the babies’ birthweights, rather than measurement. This could be an issue because recollection of birthweight can be influenced by how ill or healthy a child has been.

Exposure to landscape fire smoke frequently occurs in low-resource settings, which already have many risk factors for low birthweight. For this reason, it is important to implement policies to reduce the additional risk secondary to PM_2.5_ from landscape fires. Such policies include the promotion of alternative agricultural practices to reduce deforestation and burning of crop residue; better forest management to decrease fuel buildup; and climate change mitigation actions that incentivize clean transportation and renewable power generation. According to Li et al.’s results, these measures could help mitigate low birthweights due to PM_2.5_ in low to middle-income countries where these particles are mostly released by landscape fires, rather than other types of air pollution.
